# Supplementary material for: Pre-fusion RSV F strongly boosts pre-fusion specific neutralizing responses in cattle pre-exposed to bovine RSV
Source: Nat Commun. 2017 Oct 20;8:1085. doi: 10.1038/s41467-017-01092-4 (PMC5651886; doi:10.1038/s41467-017-01092-4)
Supplement: Supplementary file 1 — Supplementary Information [file 41467_2017_1092_MOESM1_ESM.pdf]

a

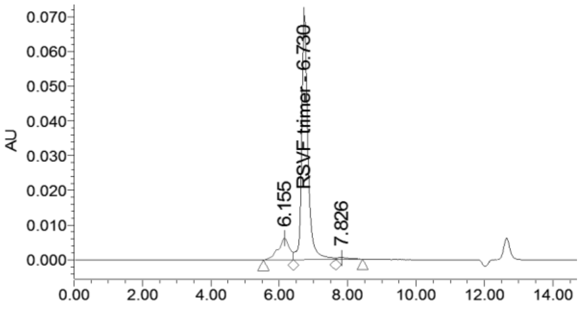

b

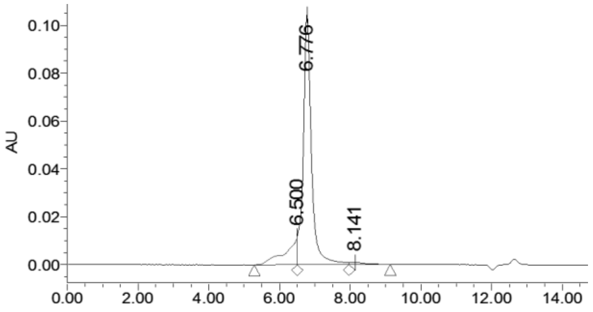

Supplementary Figure 1. Biochemical characterization of the PostF antigen. Ultra performance liquid chromatography (UPLC) traces for post F (**a**) and DS-CAV1 (**b**), respectively, on a BEH200 SEC column connected to a Waters Acquity UPLC system.

a

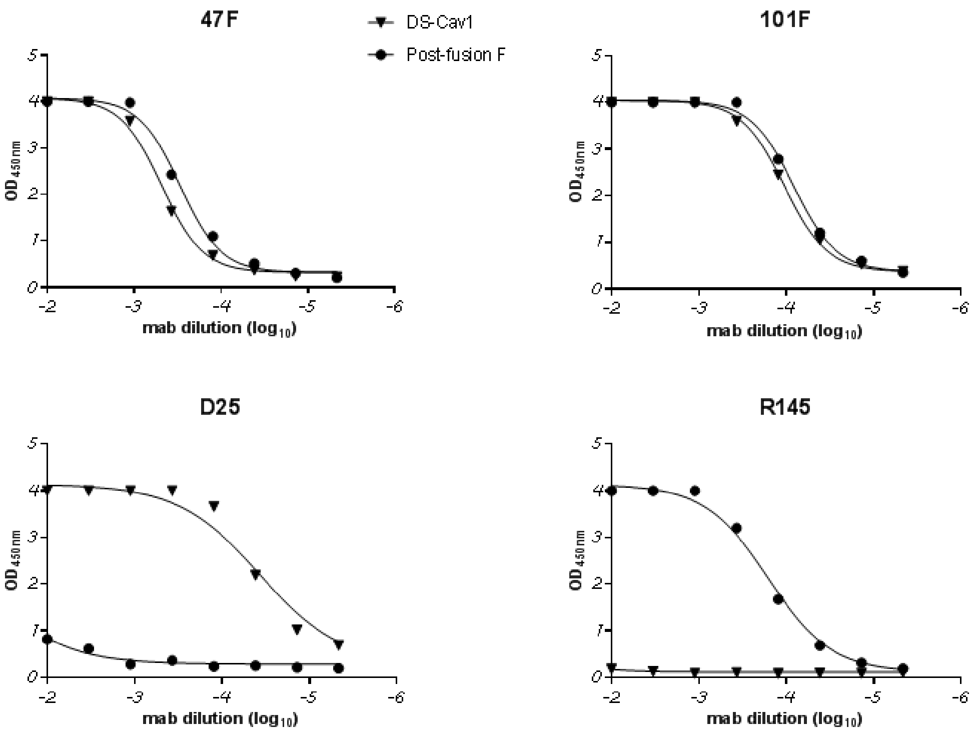

b

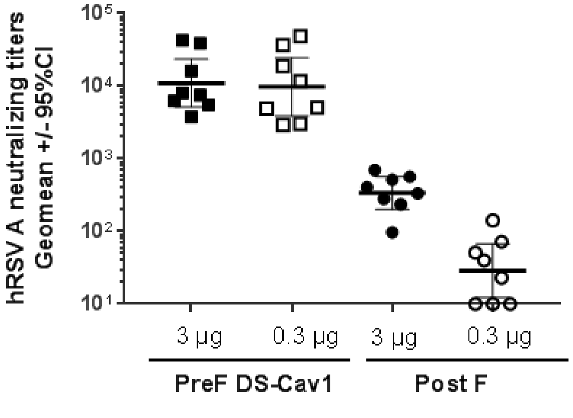

Supplementary Figure 2. Antigenic and immunogenic characterization of antigens. (**a**) Direct ELISA binding data with 47F, 101F, D25 and R145 monoclonal antibodies. 47F and 101F are antibodies recognizing both pre-fusion and post-fusion forms of F, D25 is a pre-fusion-specific antibody and R145 an antibody recognizing the 6-helix bundle existing only on the post-fusion form. (**b**) hRSV neutralizing antibody titers in mouse serum, 14 days after 2 administrations (3 weeks apart) of 2 µg of MF59-adjuvanted DS-Cav1 or Post F. Doses of protein are indicated on the X-axis. Horizontal bars indicate geometric mean titers and error bars 95% confidence intervals.

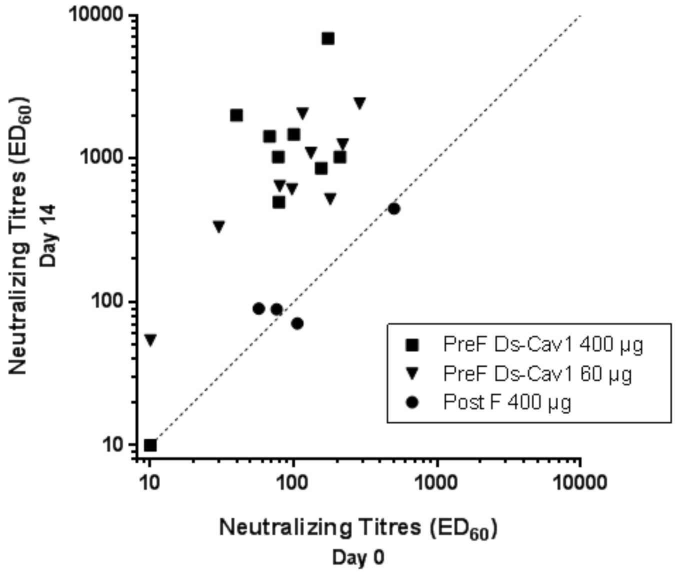

Supplementary Figure 3. Relationship between Day 0 and Day 14 hRSV neutralization titers for individual animals.

a

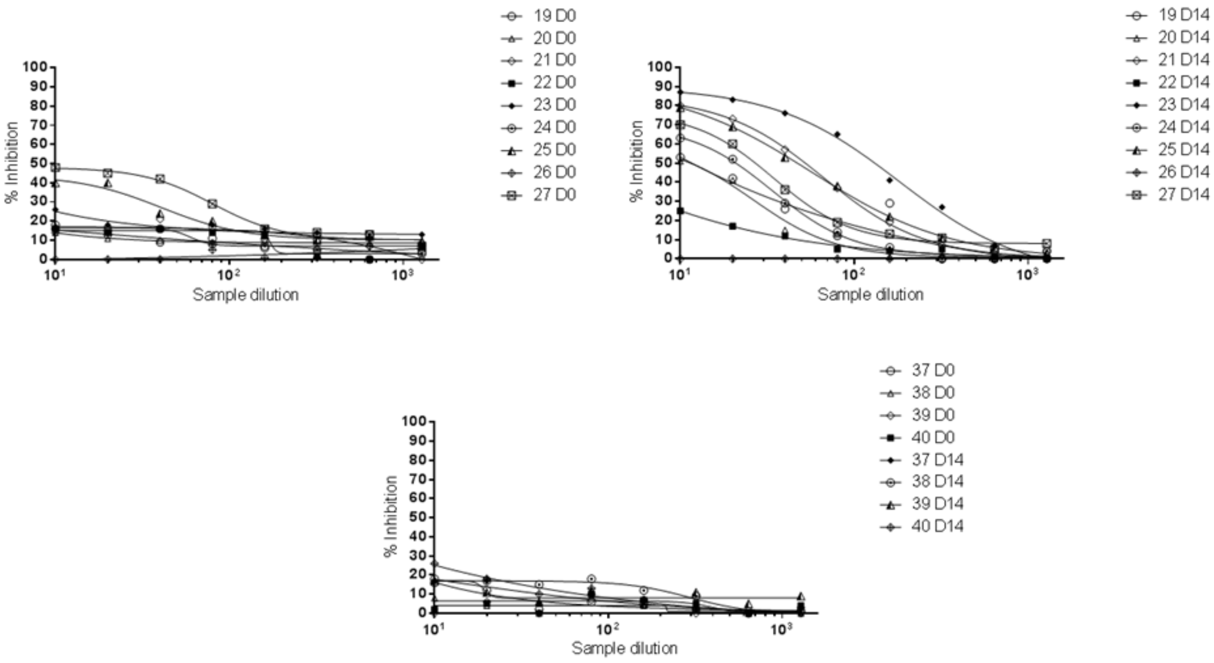

b

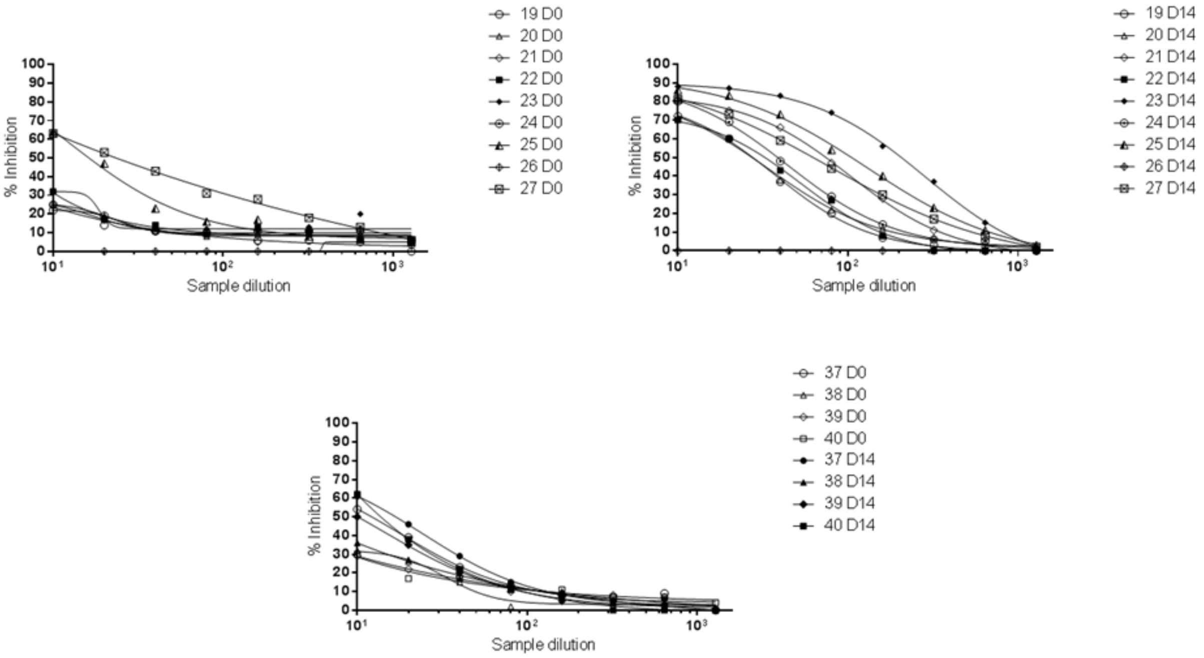

Supplementary Figure 4. Individual inhibition curves for the D25- (**a**) and Palivizumab- (**b**) competition assays. Samples for which the 1:10 dilution was above 50% inhibition had an inhibitory concentration 50% (IC50) calculated in GraphPad Prism.

		Neutralization titers								
		hRSV A Long					hRSV B		bRSV	
Group	Animal #	Day 0	Day 14	Day 28	Day 42	Day 56	Day 0	Day 14	Day 0	Day 14
400 µg Ds-Cav1	19	68	1430	1270	1049	1113	93	1610	3787	31080
	20	78	1030	376	514	435	117	1627	2015	9520
	21	40	2000	1223	1171	891	28	3144	2285	21417
	22	156	857	660	313	258	191	1260	6438	9540
	23	173	6876	4268	4050	3182	189	7612	2305	128000
	24	79	499	833	575	425	201	1508	6993	18860
	25	100	1470	883	587	536	208	9631	1519	47433
	26*	10	10	10	10	10	Neg	6	100	200
	27	210	1024	1598	307	826	300	1745	13800	97300
	**GMT**	**99**	**1378**	**1086**	**733**	**713**	**139**	**2586**	**3736**	**30099**
60 µg Ds-Cav1	28	180	521	523	451	338	360	1727	5505	38283
	29	288	2412	3229	1248	975	327	2130	6412	85547
	30	220	1251	794	1158	779	414	992	13969	17171
	31	80	642	427	350	156	121	869	3206	10600
	32	132	1094	1155	985	1199	377	1266	5952	22200
	33	30	333	119	162	45	37	295	1753	5108
	34	115	2056	1016	602	545	105	611	12416	103762
	35	97	611	992	639	618	163	1739	6706	14109
	36	10	54	37	38	25	33	202	1393	2480
	**GMT**	**90**	**664**	**523**	**433**	**297**	**153**	**859**	**4988**	**18431**
400 µg Post F	37	500	448	383	273	216	602	596	2410	3533
	38	106	71	68	70	47	66	151	4325	5374
	39	76	89	111	71	107	167	136	11351	12514
	40	57	90	70	86	41	114	215	2469	2195
	**GMT**	**123**	**126**	**119**	**104**	**82**	**166**	**227**	**4134**	**4779**

Supplementary Table 1. Individual human/bovine RSV neutralizing antibody titers. GMT: Geometric mean titer

* Animal negative for hRSV A, hRSV B and bRSV neutralization on Day 0 and did not seroconvert. Excluded from the GMT and geometric mean ratios calculations.

Group	Animal #	bRSV IgG				
		Day 0	Day 14	Day 28	Day 42	Day 56
400 µg Ds-Cav1	19	+++	++++	++++	++++	++++
	20	+	+++	++++	+++	++++
	21	++	++++	++++	++++	++++
	22	+++	++++	++++	++++	++++
	23	+++	++++	++++	+++++	++++
	24	++	++++	++++	++++	++++
	25	++	+++	++++	++++	++++
	26*	0	0	0	0	0
	27	++	+++	++++	++++	++++
60 µg Ds-Cav1	28	+++	++++	++++	++++	++++
	29	+++	++++	++++	++++	++++
	30	+++	++++	++++	++++	++++
	31	++	+++	+++	+++	+++
	32	++	++++	++++	++++	++++
	33	++	+++	+++	+++	+++
	34	+++	+++	++++	++++	+++
	35	+++	++++	++++	++++	++++
	36	+	+++	+++	++	++
400 µg Post F	37	+++	++++	++++	++++	++++
	38	++	+++	+++	+++	+++
	39	++	+++	++++	++++	++++
	40	+++	++++	++++	++++	++++

Supplementary Table 2. Individual bRSV-specific IgG semi-quantitative results.

* Animal negative for hRSV and bRSV neutralization on Day 0 and did not seroconvert.
